# On Modern Progress in Ophthalmic Medicine and Surgery

**Published:** 1894-01-27

**Authors:** Robert Brudenell Carter

**Affiliations:** Consulting Ophthalmic Surgeon to St. George's Hospital


### ON MODERN PROGRESS IN OPHTHALMIC MEDICINE AND SURGERY.

By ROBERT BRUDENELL CARTER, F.R.C.S., Consulting Ophthalmic Surgeon to St. George's Hospital.

THE preceding sketch of the rise and progress of our knowledge respecting glaucoma may perhaps be fitly followed by an account of the oncoming of the malady as it is now recognized by surgeons, and of the measures which are taken for its relief. We have seen that it essentially consists of a disturbance of the normal balance between secretion within the eye and transudation through its tunics, a disturbance of such a kind that the fluid contents of the organ come to be present in excess, and to exert injurious pressure upon parts which it is their proper office to sustain and protect. We may consider the effects of this pressure as it is exerted, first, upon the nerves, secondly, upon the blood vessels, and, lastly, in altering the relative positions of structures within the globe. These effects are, of course, produced simultaneously; but they are most conveniently described in the foregoing order.

The nerves of the eye consist of the optic nerve, which pierces the external tunics by a single opening about a millimetre and a half in diameter, the centre of which is situate nearly on the horizontal meridian, and about four millimetres to the nasal side of the posterior pole of the eyeball, and of the long and short ciliary nerves, twelve or fifteen in number, fine filaments which pass through a circle of small perforations ranged in a circle around the optic nerve, and a short distance away from it. The optic nerve administers to the sense of sight alone, and its constituent fibres are spread out into a fine membrane, called the retina, which lines the posterior two-thirds of the eyeball, and receives visual impressions which are transmitted to the brain. The central portion of the retina is the most sensitive to these impressions, and the sensibility regularly diminishes towards the periphery, with the result that, when the eye is directed to any point, the impression derived from that point falls on the centre of the retina and is seen distinctly, while at the same time there is a less distinct vision of a tolerably wide ellipsoidal space around the point looked at, a space which is called the field of vision, and which, for each eye singly, covers about 120 degrees from above downwards, and about 160 degrees from side to side. The long ciliary nerves are derived from the third nerve, and are motor only. They pass forward in slight grooves chanelled in the inner side of the sclera, to reach the ciliary processes, where they split up for distribution to the ciliary muscle and to the muscle of the iris which produces contraction of the pupil. The short ciliary nerves contain motor filaments from the third, but they also contain sensory filaments from the fifth, and filaments from the sympathetic which govern the diameter of bloodvessels, and which may also be concerned in producing dilatation of the pupil. Compression of the long and short ciliary nerves, especially if it be at first gentle and gradually increased, brings about an equally gradual impairment and loss of their activity. Their most noticeable, if not their most important function, is that of adjusting the eye, by increased convexity of the crystalline lens, for the distinct vision of small and near objects, as in reading. The power of accomplishing this adjustment becomes impaired with advancing life, the impairment constituting what is called presbyopia; but it is then due to increased hardness of the lens, which yields imperfectly to muscular action. A similar impairment attends the early stages of glaucoma, but is a consequence of diminished energy of nerve function, so that the required action upon the lens is less powerfully exerted. The increase of glaucomatous presbyopia is more rapid than that of normal presbyopia; and so, when a person who requires convex spectacles for reading finds that stronger and stronger ones become necessary at short intervals, the approach of glaucoma may be suspected. This, as a matter of fact, from which they formed very erroneous inferences, has long been known to spectacle sellers. They observed that customers of middle age, who came frequently for stronger and stronger convex glasses were apt, before long, to become totally blind; and they jumped to the conclusion that strong glasses were injurious, instead of recognising that the demand for them was only a symptom of a disease which at one time always terminated in loss of sight. The failure of motor power under the pressure of glaucoma is further shown by impaired motility of the pupil, which tends to be somewhat dilated, and is sluggish in its movements; while the participation of the sensory filaments of the short ciliary nerves is shown by diminished sensitiveness of the eye surface, which can be lightly touched with the finger tip or with a feather without provoking reflex closure of the lids. At the same time the pressure on the retina tends gradually to diminish its sensibility to visual impressions, and that in a direction from the periphery towards the centre, so that the field of vision becomes smaller; the change being usually most obvious on the nasal side of the eye under consideration. The comparatively large surface of entrance of the optic nerve forms the weakest and most yielding part of the interior of the eye, and hence it gradually gives way to the pressure exerted upon it, and becomes sunken or excavated, the nerve being, in fact, partly pushed out of the globe by the increasing amount of fluid. We have, therefore, as symptoms of pressure upon nerves within the eye, an abnormal and increasing presbyopia, a diminution of surface sensibility, a sluggishness with tendency to dilatation of the pupil, a contraction of the field of vision, and, discoverable only by the aid of the ophthalmoscope, an excavation or "cupping" of the surface of the optic nerve. The pressure upon bloodvessels has, for its first effect, to dilate a series of small veins which emerge from the front of the eye through perforations in the sclera, and pass away under the conjunctiva. These veins, which are not part of the conjunctival system, are either invisible or inconspicuous in healthy conditions, but the trunks of the venæ vorticosæ, the main channels of exit for blood from the eyeball, which pierce the sclera behind the equator, pierce it so obliquely that they are soon compressed by increasing tension, and the blood which they would naturally convey is compelled to find other ways of escape. These are furnished, more or less imperfectly, by the anterior veins already mentioned, which become not only dilated by dark blood and plainly visible, but usually tortuous. In the meanwhile, the fulness of the eye renders the entrance of the ordinary supply of arterial blood difficult, and only possible at the acme of the pulse wave, when it forces its way by pushing back some of the venous blood which is seeking exit through the central vein of the retina. This pushing back of venous blood occasions an ophthalmoscopic appearance of pulsation in the veins upon the nerve surface. often followed, as tension continues to increase, by similar pulsation in the arteries.

